# Unexpected Antioxidant Efficiency of Chlorogenic Acid Phenolipids in Fish Oil-in-Water Nanoemulsions: An Example of How Relatively Low Interfacial Concentrations Can Make Antioxidants to Be Inefficient

**DOI:** 10.3390/molecules27030861

**Published:** 2022-01-27

**Authors:** Marlene Costa, Sonia Losada-Barreiro, António Vicente, Carlos Bravo-Díaz, Fátima Paiva-Martins

**Affiliations:** 1 REQUIMTE-LAQV, Department of Chemistry and Biochemistry, Faculty of Sciences, University of Porto, 4169-007 Porto, Portugal; marlene.andreia.costa@gmail.com (M.C.); sonia@uvigo.es (S.L.-B.); 2 Department of Physical Chemistry, Faculty of Chemistry, Universidade de Vigo, 36200 Vigo, Spain; cbravo@uvigo.es; 3 Centre of Biological Engineering, Campus de Gualtar, University of Minho, 4710-057 Braga, Portugal; avicente@deb.uminho.pt

**Keywords:** chlorogenic acid, emulsion, nanoemulsion, antioxidants, distribution, lipid oxidation, pseudophase kinetic model, phenolipids, droplet size, interfacial concentration

## Abstract

Selecting effective antioxidants is challenging since their efficiency in inhibiting lipid oxidation depends on the rate constants of the chemical reactions involved and their concentration at the reaction site, i.e., at the interfacial region. Accumulation of antioxidants at the interface of emulsions is key to modulate their efficiency in inhibiting lipid oxidation but its control was not well understood, especially in emulsions. It can be optimized by modifying the physicochemical properties of antioxidants or the environmental conditions. In this work, we analyze the effects of surfactant concentration, droplet size, and oil to water ratio on the effective interfacial concentration of a set of chlorogenic acid (CGA) esters in fish oil-in-water (O/W) emulsions and nanoemulsions and on their antioxidant efficiency. A well-established pseudophase kinetic model is used to determine in the intact emulsified systems the effective concentrations of the antioxidants (AOs). The relative oxidative stability of the emulsions is assessed by monitoring the formation of primary oxidation products with time. Results show that the concentration of all AOs at the interfacial region is much higher (20–90 fold) than the stoichiometric one but is much lower than those of other phenolipid series such as caffeic or hydroxytyrosol derivatives. The main parameter controlling the interfacial concentration of antioxidants is the surfactant volume fraction, Φ_I_, followed by the O/W ratio. Changes in the droplet sizes (emulsions and nanoemulsions) have no influence on the interfacial concentrations. Despite the high radical scavenging capacity of CGA derivatives and their being concentrated at the interfacial region, the investigated AOs do not show a significant effect in inhibiting lipid oxidation in contrast with what is observed using other series of homologous antioxidants with similar reactivity. Results are tentatively interpreted in terms of the relatively low interfacial concentrations of the antioxidants, which may not be high enough to make the rate of the inhibition reaction faster than the rate of radical propagation.

## 1. Introduction

Polyunsaturated fatty acids (PUFAs) are an essential part of parenteral nutrition, which is considered a lifesaving therapy [1,2]. Patients with polyunsaturated fatty acids (PUFAs) [3] deficiency develop several diseases and show growth impairment because the human organism cannot synthesize the essential fatty acids linoleic and linolenic acids and also has a limited capacity to biosynthesize long chain fatty acids from linolenic acid. In recent years, there was great interest in using fish oils in food products mainly because of the high content of long chain omega-3 PUFAs present in their triacylglycerols, especially docosahexaenoic acid (DHA, C22: 6, ⍵-3) and eicosapentaenoic acid (EPA, C20: 5, ⍵-3) [4,5,6]. These omega-3 PUFAs showed clear health benefits to consumers [7] and, as a consequence, incorporation of fish oil into foods has increased, above all, in countries where there is a shortage of fish and foods enriched in omega-3 [8]. However, their incorporation decreases the oxidative stability of foods because EPA and DHA are quickly oxidized. DHA and EPA have a high number of bis-allylic hydrogens, which give them a high susceptibility to form free radicals following homolytic breakdown of C-H bonds.

This oxidative degradation leads (among others) to rancidity, which represents, after microbial spoilage, the second major cause of food deterioration, leading to the rejection of rancid foods and other degraded raw materials by both consumers and industry [9,10]. On the other hand, the difficulties in preserving these foods are a major cause for the low economic viability of the use of fish species rich in fish oils. Such processes are closely related to the production of both toxic compounds that may react with other food constituents and substances that impart undesirable aromas and colors to foods [11], with a direct implication on sensory and nutritional quality, safety, lifetime, and commercial value of the products [12,13]. The problem is worsened by the fact that the oxidation reactions can be catalyzed and accelerated in the presence of metals and enzymes, and they are affected by the pH of food, storage temperature, and exposure to light [14].

Thus, delaying oxidative reactions became a major task in the food industry and research in the oxidation reactions of unsaturated lipids, and their inhibition became one of the most studied areas in food science and technology. 

**Scheme 1:**
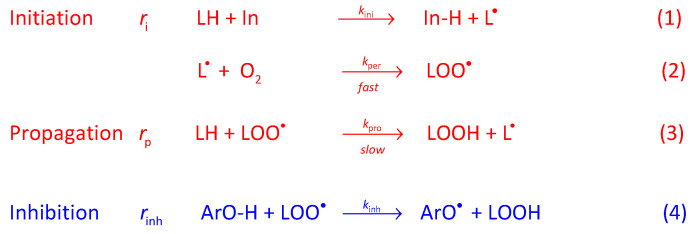
Representation of rate-limiting step of initiation and propagation steps of lipid oxidation reaction (1)–(3) and of competing inhibition reaction in presence of antioxidants (4). LH: Lipid; LOO^•^: peroxyl radical; L^•^: lipid radical; LOOH: lipid hydroperoxide; ArO-H: antioxidant and ArO^•^: antioxidant radicals.

Currently, there are several methods capable of retarding lipid oxidation: preventing access to oxygen, lowering the temperature, inactivating the enzymes that catalyze oxidation, reducing oxygen pressure, correct packaging, etc. The most effective and cheapest method of protection against lipid oxidation is undoubtedly the use of specific substances capable of inhibiting lipid oxidation reactions called antioxidants (AOs) [15,16,17,18], Scheme 1.

**Figure 1:**
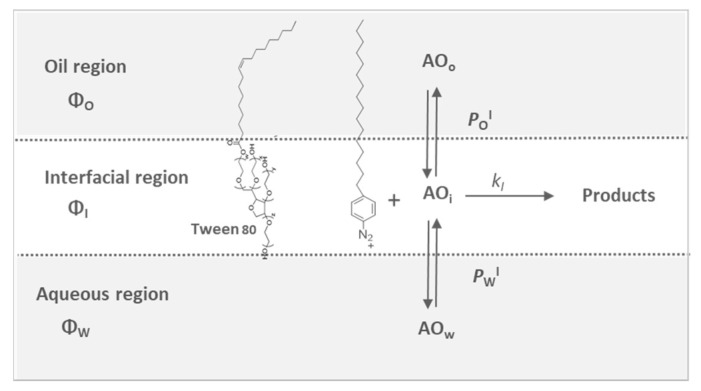
Representation of aqueous, oil, and interfacial regions of an emulsion showing approximate locations of surfactant and of reactive group of hydrophobic ArN_2_^+^ ions, as well as distribution of an antioxidant. P_O_^I^, partition constant of AO between oil-interfacial region; P_W_^I^ partition constant of AO between aqueous-interfacial region, *k*_I_, rate constant for reaction between 16-ArN_2_^+^ and AO in interfacial region. Subscripts O, I, and W indicate oil, interfacial, and aqueous regions, respectively, and Φ is volume fraction of a region (Φ = V_region_/V_total_).

Nevertheless, choosing effective AOs is challenging. The lack of scientific basis supporting the search of optimal antioxidants made it so that, to date, the finding of effective antioxidants was mainly performed based on trial and error, as the factors that affect the antioxidant efficiency and their control with AOs were not well understood, especially in emulsions. As we described in previous work [19,20,21,22,23,24], the interfacial region of emulsified systems have a foremost importance on the kinetics of the inhibition of the oxidative degradation of lipids, since the presence of the interfacial region alters the concentration of AOs (Figure 1). 

The inhibition reaction (4) in Scheme 1 occurs at the interfacial region [25,26] where the antioxidant reacts with the peroxyl radicals LOO^•^, giving LOOH and antioxidant radicals. The rate of the reaction is given by Equation (1), where parenthesis indicate real concentrations (moles per liter of interfacial region):

(1) $$r_{inh}\approx r_{inh(I)}=k_{inh(I)}{(\mathrm{LOO}}_{I}^{•}{)(\mathrm{AO}}_{I})$$

Efficient AOs are those that make the rate of inhibition higher than the rate of propagation (reaction (3) in Scheme 1), that is, *r*_*inh*_ > *r*_p_; otherwise, the production of radicals is higher than their quenching and the antioxidant is not capable of halting the oxidation reaction. As shown by Equation (1), the rate of the inhibition reaction depends on both the rate constant of the reaction with the radicals, *k*_*inh*_, (whose value depends on the chemical structure of the AO and the medium properties) and on the local concentration of the antioxidant at the reaction site. It may happen, therefore, that AOs with a high scavenging rate constant, *k*_*inh*_, are not necessarily efficient because their effective concentration may be low or not high enough to fulfill the condition *r*_*inh*_ > *r*_p_.

In this work, we want to increase our current knowledge on the factors that control the antioxidant distributions and their relationships with the antioxidant efficiency. For this purpose, we employed a series of chlorogenic acid ester derivatives of different hydrophobicity. 

Knowledge on the distribution of CGA and its derivatives and on the factors that affect their distributions may be part of promising strategies aimed at improving significantly the antioxidant efficiency and the current understanding of their effects. On the basis of the pseudophase kinetic model, we evaluated the concentration of chlorogenate esters in the interfacial region of intact fish oil-in-water emulsions and evaluated the effects of some factors that affect their distribution within emulsified systems [21].

Antioxidants’ partition between the different regions of emulsions and nanoemulsions under dynamic equilibrium conditions and their effective concentrations in each region depend on the medium or solvent properties of the various regions, but not on the size or shape of the emulsion droplets [19,27]. 

Emulsions and nanoemulsions have different droplet sizes. We recently showed that droplet sizes or interfacial areas have a negligible effect on the distributions of antioxidants and on their antioxidant efficiency [21,25,28]. Certainly, the effects of parameters that may control the distribution of AOs are not fully understood and more research is necessary to assess, for instance, what is the role of surfactant concentrations, oil-to-water ratios, and droplet sizes on the distributions of AOs and on the inhibition reactions. 

## 2. Results and Discussion

### 2.1. Partition Constants of Chlorogenic Acid and Its Esters in Binary Fish Oil-Water Mixtures, P_W_^O^

The *P*_W_^O^ values for CGA and its alkyl derivatives displayed in Table 1 reflect the lipophilic/hydrophilic nature of the compounds. The results indicate that short-chain (C1–C4) CGA derivatives are quite soluble in water. For example, in 1:1 emulsions, the number of moles of AO in the oil compared to that in water is n_AO(O)_/n_AO(W)_ = 0.2; in 4:6 or 1:9 emulsions, the ratio is even lower. Results are similar to those found in olive oil binary mixtures [22] for the same antioxidants. The high solubility in water of the short chain CGA derivatives (for instance, *P*_W_^O^_(CGA2)_ = 0.05) compared to those of hydroxytyrosol (HT, *P*_W_^O^ _(HT2)_ = 1.80) and gallic acid (GA, *P*_W_^O^ _(GA2)_ = 0.33) derivatives [22,24,29] can be attributed to the presence of the highly hydrophilic quinic acid moiety. However, chlorogenates with alkyl chains equal to or longer than 8 carbons are essentially water insoluble and their *P*_W_^O^ values could not be determined accurately.

### 2.2. Determining the Partition Constants of Chlorogenic Acid and Its Esters in Intact Fish Oil Emulsified Systems

The *P*_O_^I^ and *P*_W_^I^ values determined in fish oil emulsions and nanoemulsions for the studied chlorogenates (Section 3.6) are listed in Table 1. No significant differences were detected in the *P*_O_^I^ and *P*_W_^I^ values obtained in emulsions and nanoemulsions, with differences lower than 11%, suggesting that AO distributions are not affected by droplet sizes as demonstrated in previous works [25,26]. Calculations carried out can be found elsewhere [20,22,25,26,28,29,30,31].

*P*_O_^I^ and *P*_W_^I^ values were higher than 1 for all compounds, which means that CGA and its esters have a high affinity for the fish oil-water interface, since the Gibbs free energy is negative. However, the percentage of each compound at the interfacial region is different because the *P*_O_^I^ and *P*_W_^I^ values are different. *P*_W_^I^ values increase upon increasing the length of the alkyl chain of the CGA derivatives (hydrophobic effect) which implies that the solubility in water decreases. Thus, the CGA derivatives with longer alkyl chains such as CGA8, CGA12, and CGA16 are only distributed between the interfacial and oil regions and, therefore, their distribution is only described by the *P*_O_^I^ partition constant. The *P*_O_^I^ values of CGA8, CGA12, and CGA16 are similar, with values close to P_O_^I^ ≈ 20. These small variations are in accordance with those obtained for different phenolipids in similar emulsified systems [21,28], including gallic acid, hydroxytyrosol, caffeic and protochatechuic derivatives [20,21,28,29,32] in both emulsions, and nanoemulsions. 

The interfacial rate constants *k*_I_ for the reaction between the AOs and the probe can also be obtained from the kinetic analysis and are displayed in Table 1. *k*_I_ values are independent of the hydrophobicity of chlorogenate esters. This finding, together with the obtained similar values for the first anodic peak potential for all AOs suggest that the reactive moieties of the AOs are located in regions of similar medium properties in both nanoemulsions and coarse emulsions, Table 1 [21,24,28,33].

### 2.3. Distribution of Chlorogenic Acid and Its Esters in Fish Oil-in-Water Emulsified Systems

**Figure 2:**
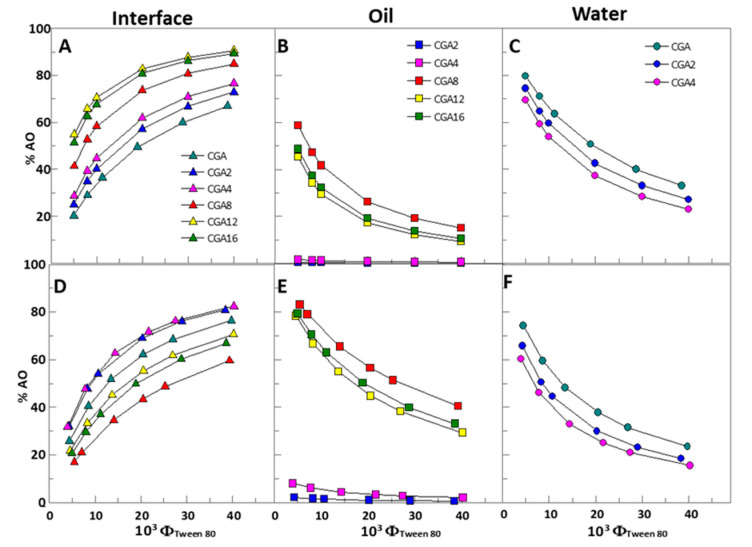
Percentages (%AO) of CGA and its esters in different regions (O—oil; I—interface; W—water) of 1:9 (O/W) nanoemulsions (**A**–**C**) and 4:6 (O/A) emulsions (**D**–**F**) (fish oil/0.04 M citrate buffer, pH = 3.65/Tween 80), T = 25 °C.

Figure 2A–C show the distribution of AOs in 1:9 O/W nanoemulsions and Figure 2D–F show the distribution of AOs in 4:6 O/W emulsions as a function of the surfactant volume fraction Φ_I_.

Figure 2A–C show that AOs were found in the interfacial region in a percentage between ~20 and 60% for an emulsifier fraction of Φ_I_ = 0.005. These values are relatively low when compared with those obtained for the hydroxytyrosol and gallic acid phenolipid series [21,24,28] in similar fish oil emulsions. The percentage of short chain AOs (up to CGA4) is quite similar to each other but somewhat smaller than those of long chain derivatives (CGA8-CGA16). Thus, at a first glance and for the sake of convenience, we will group the antioxidants in two sets: those with short alkyl chains (up to CGA4), and those with longer alkyl chains (8 or more carbon atoms). Note that AOs in the former group are essentially distributed between the aqueous and interfacial regions of emulsions and nanoemulsions, meanwhile those in the second group are mostly distributed between the oil and interfacial regions.

Results in Figure 2A–C show that, for both sets of AOs, the fraction of AO located in the interfacial region of 1:9 (O/W) nanoemulsions increases upon increasing the surfactant volume fraction, from ~20% to ~70% on going from Φ_I_ = 0.005 up to Φ_I_ = 0.04 for short chain analogs, and from ~50% up to ~80–85% for the long chain derivatives (same increase in Φ_I_). Similar variations were obtained in 4:6 O/W emulsions (Figure 2D–F) as expected from the similar partition constants obtained in both systems (Table 1). Results confirm that the fraction of emulsifier has a significant influence on the fraction of antioxidant found in each phase: the higher Φ_I_, the greater the percentage of antioxidant present in the interfacial region. Moreover, since there was an increase in the oil region and a decrease in the aqueous region volumes in the 4:6 emulsions when compared with that of the 1:9 nanoemulsions, the more hydrophilic compounds (CGA up to CGA4) increase their percentage in the interfacial region in contrast with the more lipophilic compounds, that are now in a lower percentage at the interfacial region (Figure 2D–F).

**Figure 3:**
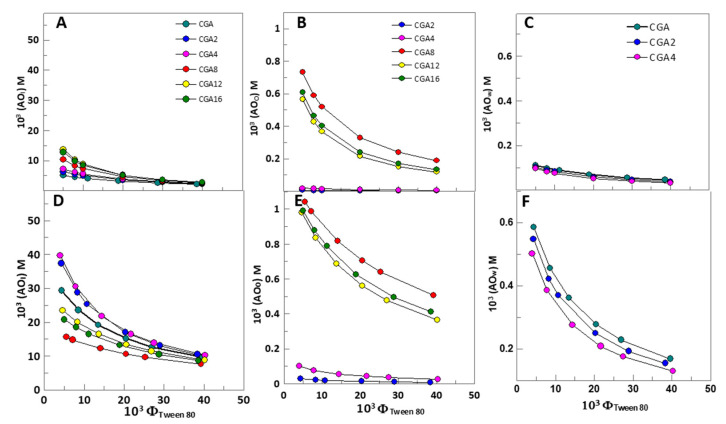
Effective concentration of chlorogenic acid (CGA) and its esters in interfacial (I), oil (O) and aqueous (W) regions of 1:9 (O/W) nanoemulsions (**A**–**C**); [AO_T_] = 0.125 mM) and 4:6 (O/W) emulsions (**D**–**F**); [AO_T_] = 0.500 mM).

As indicated in Equation (1), the rate of the inhibition reaction by AOs depends on the effective concentration of the reactants at the reaction site [20,21,22,24,28,30,34]. The effective AOs concentration in each region of the emulsified system can be distinct from the stoichiometric concentration [AO_T_] because AOs are distributed in the different regions of the emulsified system which have different volumes. For this reason, it is important to gain knowledge on the actual values of the concentration of CGA and its esters in the different regions of fish oil-in-water nanoemulsions (1:9) and emulsions (4:6). The concentrations, expressed in moles per liter of volume of the particular region, were estimated as indicated in Section 3.6, and Figure 3 shows their variations with Φ_I_. 

**Figure 4:**
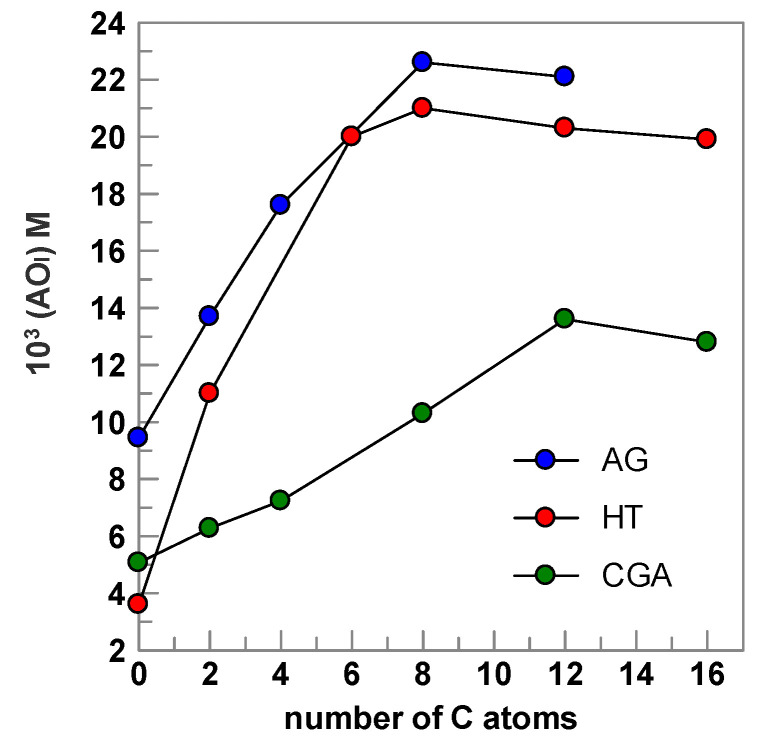
Interfacial concentration (AO_I_) for different phenolipids series in 1:9 (O/W) nanoemulsions ([AO_T_] = 0.125 mM) AG, gallic acid serie; HT, hydroxytyrosol serie. Data for AG and HT series obtained from references [21,28].

As can be observed in Figure 3A–C, the effective AO concentrations of the AOs are higher or lower than the stoichiometric concentration, [AO_T_], because of the different volumes of each region and the different distribution of the AOs (Figure 2). At any Φ_I_, the effective concentration of AOs in the interfacial region is much higher, 17–95-fold, than the stoichiometric concentration, [AO_T_]. In spite of being (AO_I_) values for CGA derivatives much higher than [AO_T_], the effective concentrations are about half of those obtained in previous studies in a similar emulsified system for hydroxytyrosol and gallic acid esters (20–170- and 20–190-fold, respectively) [21,28] as illustrated in Figure 4.

The effective concentrations of CGA2 and CGA4 derivatives in the aqueous region are 1–3 times lower than the stoichiometric concentration, but it is at least around three times higher than those reported for the hydroxytyrosol and gallic acid derivatives with similar alkyl chains [21,28]. On the other hand, the effective concentration in the oil region of the most lipophilic compounds (CGA8–CGA16) is up to five times higher than the stoichiometric concentration. 

Note the dilution effect observed for all CGA derivatives upon increasing the surfactant volume fraction; on going from Φ_I_ = 0.0045 up to 0.045, the effective concentration of AOs decreases up to 4-fold in the interfacial region, up to 6-fold in the oil region, and up to 2.5-fold in the aqueous region. The reason for the decrease in the oil and aqueous regions is because of the decrease in the percentage of AOs in this region upon increasing Φ_I_, Figure 2. The decrease in the effective concentration at the interfacial region is a consequence of two opposite effects: on one hand, the fraction of AOs in the interfacial region increases upon increasing Φ_I_ but, on the other hand, the interfacial volume increases, and the increase in the percentage of AO does not compensate the increase in the interfacial volume, resulting in an effective dilution of the antioxidants. 

### 2.4. Effects of the Oil to Water Ratio, Droplet Size, and the Emulsifier Volume Fraction on the Effective Interfacial Concentrations of Chlorogenic Acid and Its Esters in Fish Oil Emulsified Systems

**Figure 5:**
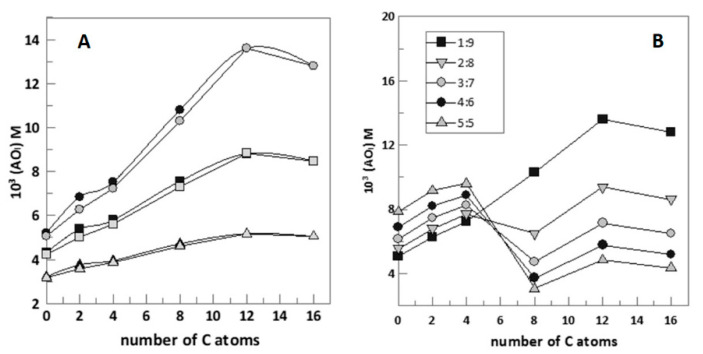
(**A**) Interfacial concentrations of CGA and CGA esters in 1:9 (O/W) emulsions (●—Φ_I_ = 0.005, ■—Φ_I_ = 0.01, ▲—Φ_I_ = 0.02) and 1:9 (O/W) nanoemulsions (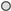
—Φ_I_ = 0.005, 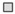
—Φ_I_ = 0.01, 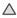
—Φ_I_ = 0.02) at different emulsifier volume fractions. (**B**) Effect of O/W ratio on interfacial AOs concentration in nanoemulsions at Φ_I_ = 0.005. In both graphs, [AO_T_] = 0.125 mM.

We demonstrated in previous works that there is a positive correlation between the interfacial antioxidant concentrations and their antioxidant efficiency [20,21,24,29]. Thus, it is interesting to modulate the antioxidant concentrations in the different regions of the emulsified systems, and for this purpose, we analyzed the effects of the oil to water ratio, droplet size, and the surfactant volume fraction employed in the preparation of the emulsion on the effective interfacial concentrations, Figure 5. 

The prepared emulsions and nanoemulsions have different droplet sizes, and despite this, similar trends were obtained for the effects of Φ_I_ on the effective interfacial concentrations of 1:9 O/W emulsified systems, as shown in Figure 5A, demonstrating that droplet size has no influence on the distributions of CGA and its derivatives. 

Figure 5B shows the important but complex influence of the O/W ratio employed in the preparation of emulsions and nanoemulsions on the interfacial concentrations of AOs (Φ_I_ = 0.005). The impact of the O/W ratio on the interfacial concentration depends on the hydrophobicity of the AO: the hydrophilic AOs (CGA–CGA4) increase upon increasing the O/W ratio (that is, with decreasing of the volume of the aqueous region). In contrast, the concentration of the most hydrophobic AOs (CGA8–CGA16) decreases upon increasing Φ_W_. For example, for a stoichiometric concentration [AO_T_] = 0.125 mM, (CGA_I_) increases from ~5 mM to ~8 mM upon going from Φ_W_ = 0.9 to Φ_W_ = 0.6 but the effective concentration of CGA12 in the interfacial region, (CGA12_I_) decreases from ~12 mM to ~4 mM when the O/W ratio changes from 1:9 to 5:5. Therefore, upon changing the O/W ratio we can modulate the relative concentration of the AOs, so that for 1:9 emulsions the AO with the highest concentration in the interfacial region is the CGA12 derivative (~12 mM), but for 3:7, 4:6, and 5:5 emulsions the AO with the highest concentration in the interfacial region is CGA4. 

These results demonstrate, therefore, that it is possible to significantly modulate the AO concentrations in the interfacial region by changing the O/W ratio, as well as to change the relative order of the concentration of antioxidants in the interfacial region: an increase in the O/W ratio promotes the incorporation of hydrophilic AOs in the interfacial region of the emulsions but decreases the incorporation of hydrophobic AOs.

### 2.5. Antioxidant Efficiency of Chlorogenic Acid and Its Esters in Fish Oil Emulsified Systems

**Figure 6:**
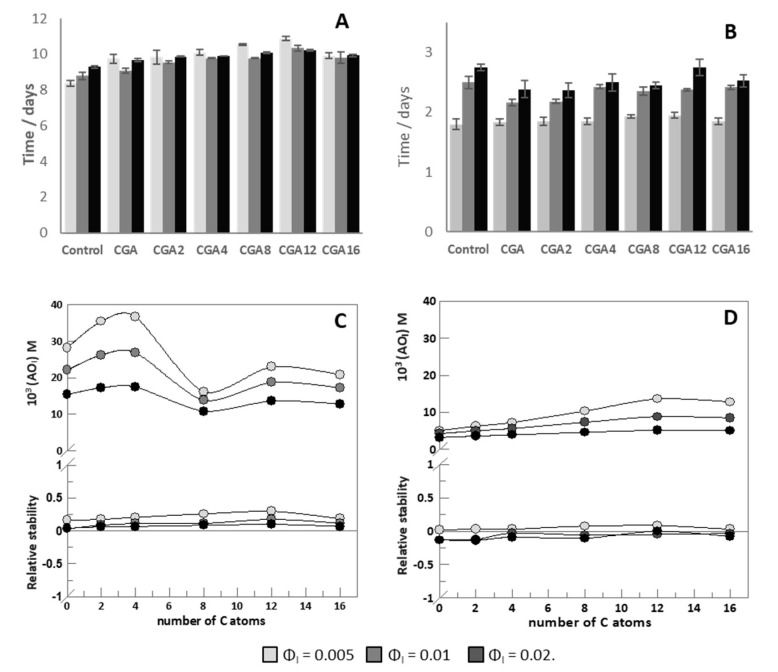
Effects of AO chain length at Φ_I_ = 0.005, 0.01, and 0.02 on oxidative stability of fish oil 4:6 O/W emulsions (**A**) and 1:9 (O/W) nanoemulsions (**B**) in terms of time needed to reach an increase of 0.5% in diene conjugated content. Comparison between antioxidant efficiency of AOs in 4:6 O/A emulsions (**C**) and 1:9 O/A nanoemulsions (**D**) and values of (AO_I_) in same systems at Φ_I_ = 0.005, 0.01 and 0.02.

As in previous works, the efficiency of AOs was evaluated in 1:9 fish oil/W nanoemulsions and 4:6 fish oil/W emulsions at T = 40 °C [20,23,24], by monitoring the time necessary to reach an increase in the percentage of CDs of 0.5% in both systems, Figure 6A,B. In coarse emulsions prepared with Φ_I_ = 0.005 and 0.01, a smooth—but statistically significant—parabolic-like variation in the AO efficiency with the alkyl chain length, similar to that previously obtained in olive oil emulsions [22], was obtained. At higher surfactant volume fractions (Φ_I_ = 0.02) or in 1:9 O/W emulsions, none of the investigated compounds showed a notorious antioxidant efficiency. In previous works, the grafting of natural antioxidants with inert alkyl chains was employed as a tool to modify their hydrophobicity and substantial increases in their antioxidant efficiency were reported [21,35,36,37]. This, however, does not seem to be the case in fish oil emulsions, as envisaged from the results in Figure 6. 

According to Equation (1), the rate of the inhibition reaction depends on both the effective concentration of AOs at the reaction site (the interfacial region) [21,23,25,26,28,38] and the rate constant value for the reaction between the AOs and the lipid radicals, *k*_*inh*_. The *k*_*inh*_ values should be similar for all AOs because: (1) all the AOs employed in this work have the same reactive moiety and have similar reactivities against the model DPPH radical, (2) their first anodic potentials (Table 1) are similar, and (3) they are located in environments with similar solvent properties as demonstrated by the constancy of the *k*_I_ values for the reaction with 16-ArN_2_^+^, Table 1. Therefore, the negligible effects of AOs in inhibiting the oxidation of fish oil (poly)unsaturated lipids may be a consequence of the low interfacial concentration of AOs.

Our tentative explanation for the observed experimental behavior is based on the following rationale [39]. The oxidative stability of an emulsified system depends on the balance of several factors, including the concentration of AOs and of radicals in the interfacial region, where the inhibition reaction mainly takes place [21,25,28,31,40,41]. If the interfacial concentrations of antioxidants are low, antioxidants may not be able to remove peroxyl radicals at sufficient speed to halt the propagation step. Increases in the effective concentrations can be achieved, among others, by modifying the hydrophobicity of the antioxidants (grafting, for example, inert alkyl chains) and by modifying the environmental conditions (surfactant concentration, oil to water ratio, etc.). These alternatives were explored here but did not result in significant improvement in the antioxidant efficiency. Another possibility is to increase the amount of added antioxidant, that is, to increase the stoichiometric concentrations of the antioxidants. This possibility was not sufficiently explored despite the fact that several researchers investigated the role of the initial concentration of the antioxidants; however, in some instances, pro-oxidative effects were found and no definitive conclusions were achieved [37,42]. Indeed, addition of higher amounts of antioxidant to emulsified systems, although possible from the experimental point of view, is not always possible in practice because of health regulations. This and other possibilities are currently being explored and will be part of future reports. 

In this work, the AOs that are found in 4:6 O/W emulsions in a greater percentage in the interfacial region are the most hydrophilic AOs (CGA, CGA2 and CGA4). Thus, we would expect better antioxidant efficiency from these AOs. However, instead, we found a modestly better efficiency obtained with the more lipophilic derivatives CGA8 and CGA12. 

**Scheme 2:**
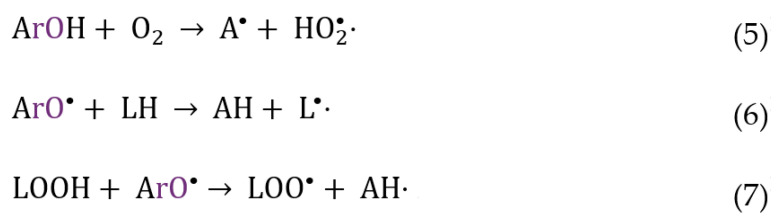
Representation of the oxidation reactions involving antioxidants (ArOH). LH: Lipid; LOO^•^: peroxyl radical; L^•^: lipid radical; LOOH: lipid hydroperoxide; ArO-H: antioxidant and ArO^•^: antioxidant radicals.

An important factor affecting the effectiveness of an antioxidant is the tendency of the radical it produces to undergo transfer with the lipid subtract, (Scheme 2), t. The effectiveness of AOs (ArOH) can thus be decreased by direct oxidation by reaction (5), and by hydrogen chain transfer reaction (6) of the AO radical (ArO**^•^**) with the lipid substrate (LH) to reinitiate the chain [40]. 

These reactions became important at high concentration of AOs and at higher temperatures. As an example, α-tocopherol can become prooxidant and chain-carriers by regenerating peroxyl radicals (LOO^•^) by reaction (7) [40].

Therefore, the effectiveness of an AO is considered as the result of the balance between the inhibition rate (*k*_*inh*_) of reaction (4) and the transfer reactions (6) and (7), meaning that its effectiveness depends on the balance between its antioxidant and its pro-oxidant activity. 

Several studies in emulsified systems reported no antioxidant effect or pro-oxidant effect from the more hydrophilic antioxidants. The impact of AO concentration in the aqueous region for the pro-oxidant effects of antioxidants, particularly in the presence of a strong chelator, is not fully understood and needs further investigation. However, the importance of the AO concentration in the aqueous region to the low oxidative stability of emulsified systems was highlighted in a recent study where an important negative correlation between AO aqueous concentration and the oxidative stability of emulsions was determined [40]. Therefore, in the 4:6 emulsion system, although the more hydrophilic AOs are a little more concentrated at the interfacial region, the balance with the pro-oxidant effect caused by the presence of the compounds in the aqueous region seems to determine the lack of antioxidant activity for these more hydrophilic compounds. Moreover, in the 1:9 O/W nanoemulsified system, where the AOs are in a lower concentration at the interfacial region, the pro-oxidant effect outweighs the antioxidant capacity of AOs located at the interfacial region and a pro-oxidant effect is observed in nanoemulsions at Φ_I_ = 0.005 containing CGA and CGA2. 

The antioxidant efficiency of most AOs decreases with the increase in the emulsifier volume [20,25,29,31,34,39]. These observations were justified by the decrease in the AOs concentration at the interfacial region due to the dilution of AOs caused by the increase in emulsifier volume (Figure 5A and Figure 6C,D). However, in this study we could observe an increase in the oxidative stability of emulsions with the increase in the emulsifier volume. Since these AOs did not show any efficiency in this nanoemulsified system, their dilution by the increase in the emulsifier volume will not affect their antioxidant capacity that was already null. In fact, a dilution of CGAs will make the rate of the inhibition reaction (reaction (4) in Scheme 1) to decrease, and hence AOs will remain inefficient. However, as discussed before, the efficiency or inefficiency of AOs also depends on the balance between the rates of the propagation and inhibition reactions and, upon increasing Φ_I_, the concentration of peroxyl radicals present in the interfacial region may decrease, reducing the value of the rate of the propagation step (reaction (3) in Scheme 1) and, therefore, leading to an increase the oxidative stability of the emulsions. However, other factors such as the decrease in the concentration of AOs in the aqueous region (Figure 3C,F) and the better coverage of droplets due to the higher amount of emulsifier (Table 2, Section 3.3) may also contribute for the higher oxidative stability observed upon the increase in the emulsifier volume in nanoemulsions without AOs or in the presence of AOs at an insufficient concentration to show antioxidant efficiency.

## 3. Materials and Methods

### 3.1. Materials

Citric acid, nonionic surfactant Tween 80 (HLB = 15.0) and N-(1-naphthyl) ethylenediamine (NED) were purchased from Acros Organics. Mili-Q grade water was employed in the preparation of emulsions. Commercial fish oil (generously provided by Biomega Natural Nutrients S.L., Boiro, A Coruña, Spain) was stripped from endogenous AOs by passing it twice through a Al_2_O_3_ column. The fish oil fatty acid composition, expressed as % total fatty acids, was 55% PUFAs containing 17% EPA and 30% DHA, 23% monounsaturated fatty acids (MUFA), and 22% saturated fatty acids (SFA). 

The synthesis and purification of chlorogenic fatty acid esters were carried out according to the procedure described by Meirelles et al. [22]. The purity of all synthetized compounds was higher than 97%. The chemical probe 4-hexadecylbenzenediazonium tetrafluoroborate, 16-ArN_2_BF_4_, was prepared according to the procedure described by Bravo–Diaz et al. [19]. 

### 3.2. Preparation of Emulsions and Nanoemulsions

Coarse emulsions were prepared by mixing stripped fish oil and Tween 80 in acidic aqueous buffer solution (0.04 M citrate buffer, pH 3.65). The volume fraction of surfactant, Φ_I_, defined hereafter as Φ_I_ = V_surf_/V_emulsion_ was varied from Φ_I_ = 0.005 up to Φ_I_ = 0.04. AOs were added in emulsifier solutions before emulsification at a final concentration of 2 mM (distribution experiments), 0.5 mM (oxidation experiments in 4:6 O/W emulsions), or 0.125 mM (oxidation experiments in 1:9 O/W nanoemulsions). The O/W mixtures were stirred at high speed with a Polytronic PT-1600 homogenizer (20,000 rpm, 1 min). 

To reduce the size of the droplets and obtain nanoemulsions with the same composition, the coarse emulsion was passed through a high-pressure homogenizer (Nozzle Z5, Nano DeBEE, Bee International, South Easton, MA, USA) at 25,000 psi (137.5 MPa) for 3 cycles. Ice bags were used to avoid the local overheating and to prevent lipid oxidation. The prepared nanoemulsions were stored in the refrigerator at T = 4 °C in the absence of light until used. To the naked eye, no phase separation was observed during storage.

### 3.3. Droplet Size and Size Distributions of Fish Oil-In Water Emulsified Systems

The droplet size and size distribution analyses were carried out using dynamic light scattering (DLS) (Zetasizer NanoZS laser diffractometer, Malvern Instruments Ltd., Worcestershire, UK) at T = 25 °C. The coarse 1:9 (O/W) emulsions prepared by employing a Polytronic PT-1600 homogenizer were highly polydisperse, with average droplet sizes of 1492 nm (Φ_I_ = 0.0047) and 506 nm Φ_I_ = 0.038). The 1:9 (O/W) nanoemulsions prepared by employing a high-pressure homogenizer showed a monomodal droplet size distribution with average droplet sizes of 311 nm (Φ_I_ = 0.0047) and 164 nm (Φ_I_ = 0.02) (Table 2). Auxiliary experiments showed that droplet sizes remain constant during the initial stage (before they became oxidized) of the oxidation experiments. Some physical characteristics of the prepared emulsions and nanoemulsions are displayed in Table 2. 

### 3.4. Antioxidant Efficiency of Chlorogenic Acid and Its Esters in Fish Oil Emulsified Systems

The antioxidant efficiency was determined as in previous works [20,21,22,24,25,28,29,31,39,43] by monitoring the formation of primary oxidation products (conjugated dienes, CDs) with time in the intact emulsified systems both in the absence (control) and in the presence of AOs. The emulsified systems were stored in an oven and allowed to oxidize spontaneously at T = 40 °C in the dark. Emulsions were vortexed for 30 s before sampling, twice a day. At selected times, aliquots (12.5 µL of emulsion, 25 µL in the case of nanoemulsions) were diluted up to 10 mL with ethanol, and the absorbance was measured at λ = 233 nm. All runs were performed in triplicate and only the average values are reported.

### 3.5. Determining the Partition Constant, P_W_^O^, of Chlorogenic Acid and Its Esters in Binary Fish Oil–Water Mixtures

*P*_W_^O^ between fish oil and citrate buffer was determined, in triplicate, by employing the same shake-flask method as in previous works [20,21,22,24,25,29,39,44] using 4:6 binary stripped fish oil and buffered aqueous solution (0.04 M citrate buffer, pH = 3.65) mixtures containing each AO at final concentrations of 3.5 mM. *P*_W_^O^ were determined by using Equation (2), where V_O_ and V_W_ are the volumes of the oil and aqueous phases, respectively.

(2) $$P_{W}^{O}=\frac{\left({\mathrm{AO}}_{O}\right)}{\left({\mathrm{AO}}_{W}\right)}=\frac{{\%\mathrm{AO}}_{o}}{{\%\mathrm{AO}}_{W}}\times \frac{V_{W}}{V_{O}}$$

### 3.6. Determining Antioxidant Distribution and Local Concentrations in Intact Fish Oil Emulsified Systems

The distribution of antioxidants in the different regions of the emulsified systems was determined by employing a chemical kinetic method, as described in previous works [19,20,21,22,24,25,29,39,45] that is based on the reduction in the chemical probe 16-ArN_2_^+^ by the AOs. The probe has its reactive group (-N_2_^+^) located exclusively in the interfacial region of the emulsions and nanoemulsions (Figure 1), where it reacts with the AOs. The experimental variations of *k*_obs_ at different Φ_I_ were analyzed according to the pseudophase kinetic model [19]. The thermodynamic partition constants of the AO between the oil-interfacial, *P*_O_^I^, and aqueous-interfacial, *P*_W_^I^, regions can be defined by Equations (3) and (4) and can be determined experimentally by fitting the variation of the observed rate constant, *k*_obs_, with Φ_I_, Equation (5).

(3) $$P_{O}^{I}=\frac{{(\mathrm{AO}}_{I})}{{(\mathrm{AO}}_{O})}$$

(4) $$P_{W}^{I}=\frac{{(\mathrm{AO}}_{I})}{{(\mathrm{AO}}_{W})}$$

(5) $$k_{\mathrm{obs}}=\frac{\left[{\mathrm{AO}}_{T}\right]k_{I}P_{W}^{I}P_{O}^{I}}{\Phi_{O}P_{W}^{I}+\Phi_{{}_{I}}P_{W}^{I}P_{O}^{I}+\Phi_{W}P_{O}^{I}}$$

In Equations (3)–(5), (AO_O_), (AO_W_), and (AO_I_) refer to the effective concentration (mol L^−1^), of AOs in the oil, aqueous and interfacial regions, respectively, and [AO_T_] stands for the stoichiometric concentration of AO in the all emulsion (mol L^−1^), *k*_I_ is the rate constant for the reaction between the AO and the probe in the interfacial region and Φ_O_, Φ_W_, and Φ_I_, are the volume fractions of oil, water and tween 80, respectively. By combining a single set of kinetic experiments (*k*_obs_ vs. Φ_I_) with the value for the partition constant between oil and water in the absence of emulsifier (*P*_W_^O^ = *P*_W_^I^/*P*_O_^I^), it is possible to calculate the values of *P*_W_^I^ and *P*_O_^I^ by solving two equations for two unknowns. Details on these determinations and simplifications employed for determining the partition constants of water-insoluble Equation (6) and oil-insoluble Equation (7) AOs in emulsified systems can be found elsewhere [20,22,25,26,28,29,32].

(6) $$k_{\mathrm{obs}}=\frac{k_{I}{\left[\mathrm{AO}\right]}_{T}P_{W}^{I}}{\Phi_{I}P_{W}^{I}{+\Phi }_{W}}$$

(7) $$k_{\mathrm{obs}}=\frac{k_{I}{\left[\mathrm{AO}\right]}_{T}P_{O}^{I}}{\Phi_{I}P_{O}^{I}{+\Phi }_{O}}$$

The percentage of AO (%AO_I_) and its effective interfacial concentration (AO_I_) in the emulsified system can be determined by using Equations (8) and (9), which involves employing the previously calculated *P*_W_^I^ and *P*_O_^I^ values.

(8) $${\%\mathrm{AO}}_{I}=\frac{100\Phi_{I}P_{W}^{I}P_{O}^{I}}{\Phi_{O}P_{W}^{I}+\Phi_{{}_{I}}P_{W}^{I}P_{O}^{I}+\Phi_{W}P_{O}^{I}}$$

(9) $${(\mathrm{AO}}_{I})=\frac{{[\mathrm{AO}}_{T}{](\%\mathrm{AO}}_{I})}{\Phi_{I}}$$

For water-insoluble and oil-insoluble AOs, the simplified Equations (10) and (11), respectively, can be applied. Details of the calculations can be found elsewhere [21,25,35].

(10) $${\%\mathrm{AO}}_{I}=\frac{{100\Phi }_{I}P_{W}^{I}}{\Phi_{I}P_{W}^{I}{+\Phi }_{W}\;}$$

(11) $${\%\mathrm{AO}}_{I}=\frac{{100\Phi }_{I}P_{O}^{I}}{\Phi_{I}P_{O}^{I}{+\Phi }_{O}\;}$$

### 3.7. Statistical Analysis

The kinetic experiments were run in triplicate for 2–3 *t*_1/2_ and *k*_obs_ values were within ±7–9%, with *r* > 0.995. CDs were determined in triplicate. SPSS 21.0 software was employed for statistical analysis by ANOVA. The significant differences among means were analyzed with Duncan’s multiple range test on a 95% confidence level (*p* ≤ 0.05). Data are displayed as mean ± standard deviation.

## 4. Conclusions

The results in this work highlight the great complexity of the oxidative phenomena and its inhibition by antioxidants (AOs) in emulsified systems, addressing some of the points that need to be considered in future studies to fully understand all the factors that contribute for the oxidative stability of emulsified systems. One of the factors affecting the AOs efficiency is their interfacial concentration, and therefore, some strategies were used to increase the interfacial concentrations of AOs by using AOs with the same reactive moiety but of different liposolubility, different surfactant volumes, or different oil/water ratios. However, for very hydrophilic and very lipophilic compounds such as the ones used in this work, these strategies were not able to increase the interfacial concentrations to a concentration high enough for AOs to remove peroxyl radicals at sufficient speed to halt the oxidative reactions and, therefore, they showed not to be efficient in preventing lipid oxidation. This is of particular importance in foods containing lipids with a high content in unsaturated fatty acids such as in the present work, where peroxyl radicals are produced at high rates. In the absence of antioxidant efficiency, other factors, such as the AO concentration in the aqueous region, that seem to contribute to the pro-oxidative activity of AOs, or the dilution of radicals at the interfacial region by the increase in the surfactant volume, became more relevant for the emulsified system’s oxidative stability. Results also showed that the interfacial concentration of AOs is not modified by the droplet size of the emulsified system. 

## Figures and Tables

**Table 1:**
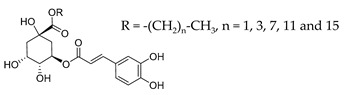
Antioxidant (AO) percentages in aqueous phase (%AO_W_) and *P*_W_^O^ values in binary fish oil-water systems. *P*_O_^I^ and *P*_W_^I^ values and rate constant in interfacial region, *k*_I_, in fish oil emulsified systems. EC50 (mole AO/mole DPPH^●^) values obtained with a level of significance *p* < 0.05 and anodic potential *E*_pa_ versus Ag–AgCl measured at a glassy carbon electrode for 10^−4^ mol L^−1^ solutions of compounds in buffer solution in absence and presence of 2% Tween 80.

Compound		CGA	CGA2	CGA4	CGA8	CGA12	CGA16
**Binary** **System**	(%AO_W_)	99.4 ± 0.15	97.1 ± 0.52	87.5 ± 0.53	≈ 0	≈ 0	≈ 0
	*P*_W_^O^	0.009 ± 0.002	0.05 ± 0.01	0.2 ± 0.01	---	---	---
**Emulsion** **O/W**	*P*_W_^I^	47 ± 3	67 ± 5	78 ± 9	---	---	---
	*P*_O_^I^	---	1344 ± 94	390 ± 44	15 ± 7	24 ± 3	21 ± 5
	10^2^ *k*_I_ (M^−1^s^−1^)	1.9 ± 0.7	2.8 ± 0.4	3.6 ± 0.7	3.7 ± 0.1	3.4 ± 0.1	3.5 ± 0.1
**Nanoemulsion** **O/W**	*P*_W_^I^	46 ± 3	60 ± 8	74 ± 5	---	---	---
	*P*_O_^I^	---	1195 ± 166	369 ± 26	14 ± 1	24 ± 4	21 ± 3
	10^2^ *k*_I_ (M^−1^s^−1^)	4.4 ± 0.1	5.5 ± 1.2	7.7 ± 0.9	6.8 ± 0.1	7.2 ± 0.1	6.4 ± 0.1
EC_50_ * ± σ^a^	5 min	0.243 ± 0.004	0.243 ± 0.003	0.248 ± 0.003	0.244 ± 0.003	0.249 ± 0.006	0.237 ± 0.006
	15 min	0.235 ± 0.005	0.217 ± 0.005	0.234 ± 0.002	0.234 ± 0.004	0.238 ± 0.004	0.223 ± 0.006
	60 min	0.234 ± 0.006	0.172 ± 0.012	0.179 ± 0.005	0.184 ± 0.008	0.189 ± 0.005	0.161 ± 0.005
*E*_pa_ * (V)	0% Tween 80	0.391	0.410	0.397	0.406	0.398	0.390
	2% Tween 80	0.398	0.403	0.400	0.409	0.407	0.400

* Data extracted from Meireles et al. [22].

**Table 2:** Theoretical calculations of some physical characteristics of prepared oil-in-water (O/W) fish nanoemulsions and emulsions under different experimental conditions. d = droplet diameter; V_droplet_ = volume of one droplet; S_d_ = droplet surface; N_d_ = total number of droplets; V_total, oil_ = total volume of oil per 100 g of emulsion; S_tota_l = surface of all droplets; m_T80, droplet_ = mass of surfactant required for saturation per 100 g of emulsion (calculated by employing an interfacial coverage at saturation of Г∞ = 2 × 10^−6^ (mol m^−2^); m_T80_, _residual_ = residual mass of Tween 80 remaining in aqueous phase (mT80, mass of Tween 80 used).

	Nanoemulsions			Emulsions		
**Φ_o_**	1.0	1.0	1.0	4.0	4.0	4.0
**10^2^ Φ_I_**	**0.5**	**1.0**	**2.0**	**0.5**	**1.0**	**2.0**
**V-potential (mV)**	−18.1	−14.2	−13.5	−22.6	nd	nd
**10^6^ d (m)**	0.311	0.243	0.164	4.82	3.08	2.66
**10^12^ S** **_droplet_ (m^2^)**	0.30	0.18	0.09	71.3	30.2	22.8
**10^20^ V** **_droplet_ (m^3^)**	1.51	0.69	0.24	5343	1610	1038
**10^−12^ N_d_**	66.5	147	405	0.75	2.43	3.66
**S_total_ (m^2^)**	19.2	24.7	36.5	4.98	7.80	8.57
**10^2^ m** **_T80,droplet_ (g)**	0.51	0.65	0.96	0.13	0.20	0.22
**10^2^ m** **_T80,excess_ (g)**	−0.01	0.35	1.04	0.37	0.80	1.78

## Data Availability

Not applicable.

## Sample Availability

Samples of the compounds are not available.
